# Editorial: Immunobiology and immunotherapeutics in myelodysplastic syndrome and acute myeloid leukemia

**DOI:** 10.3389/fimmu.2024.1500864

**Published:** 2024-10-16

**Authors:** Shyam A. Patel, Alessandro Isidori, Claudio Cerchione, Anna Aureli

**Affiliations:** ^1^ Dept. of Medicine – Division of Hematology/Oncology, Center for Clinical and Translational Science, Morningside Graduate School of Biomedical Sciences, UMass Chan Medical School, Worcester, MA, United States; ^2^ Haematology and Stem Cell Transplant Center, Azienda Ospedaliera Ospedali Riuniti Marche Nord (AORMN), Pesaro, Italy; ^3^ Hematology Unit, IRCCS Istituto Scientifico Romagnolo per lo Studio e la Cura dei Tumori (IRST), Meldola, Italy; ^4^ Institute of Translational Pharmacology, National Research Council of Italy, L’Aquila, Italy

**Keywords:** myelodysplastic syndromes, acute myeloid leukemia, immunotherapy, leukemia, myeloid neoplasia

Myelodysplastic neoplasms (MDS) and acute myeloid leukemia (AML) are hematologic malignancies often characterized by cytopenias and ineffective erythropoiesis due to presence of a genomically aberrant hematopoietic stem/progenitor pool. The pathogenesis and ontogeny of MDS and AML involves mutated hematopoietic stem cells that inevitably undergo clonal evolution, leading to various clinical manifestations such as fatigue, infections, and bleeding. MDS and AML have historically been defined as two distinct diseases, but contemporary literature suggests that they may lie on the same continuum of myeloid neoplasms. The disease biology of MDS and AML is critical to understand, given that molecular target validation and therapeutic development often stems from a deep understanding of the genomics and proteomics of these diseases.

The advent of next-generation sequencing (NGS) has led to transformative benefits in terms of MDS and AML therapeutics in the recent 5-10 years, though much work remains to be done to help address the unmet needs that these diseases pose. To date, only a handful of molecular targeted, precision therapeutics have been granted approval by the U.S. Food & Drug Administration or the European Medicines Agency. In the recent years, attempts at harnessing autologous immune surveillance mechanisms have been made for MDS and AML (1).

In this Research Topic in *Frontiers in Immunology*, we present a series of impactful basic, clinical, and translational studies at the cutting edge of immunobiology of MDS and AML. We summarize the major findings of these articles herein and offer our perspectives on how these findings can help advance the field.

Rezaei et al. performed a multi-institutional collaboration to study the effects of T cell immunoglobulin domain and mucin (TIM-3)/Gal-9 interaction on the metabolomics of AML. Using 2 different AML cell lines, the authors measured mRNA expression of a variety of glucose and lipid metabolism regulators as well as AML cell proliferation and survival. They found significant elevation of extracellular free fatty acids, glucose consumption, and concentration of glutathione in the presence of Gal-9 (2). The interaction between Tim-3 and Gal-9 appears to protect cells from oxidative damage, which in turns results in enhanced AML cell proliferation. The study by Rezaei et al. had shed important translational insight. Early this year, the STIMULUS-MDS1 trial by Zeidan et al. reported the results of hypomethylating agents in combination with the anti-TIM-3 antibody sabatolimab (2). Complete response rate was 22% and median progression-free survival was 17.8 months. A randomized Phase 3 trial is ongoing, and TIM-3/Gal-9 blockade may offer promising therapeutic potential. Biomarker studies, such as TIM-3 expression on individual patient samples, may help predict the likelihood of achieving responses on a personalized basis.

The study by Zhang et al. from the Central People’s Hospital of Zhanjiang assessed a new prognostication system in AML based on a specific epigenetic modification. The authors explored raw data from The Cancer Genome Atlas (TCGA), Gene Expression Omnibus (GEO), and TARGET datasets and used consensus clustering to classify patients with AML into 3 groups based on 7-methylguanosine signatures. They screened 6 genes and developed a risk-score model that could predict survival outcomes in AML. The results by Zhang et al. have important implications in biomarker-based prognostication in AML, especially given the high genetic heterogeneity across AML. Validation of epigenetic-based prognostic biomarkers may help further refine contemporary prognostication approaches, such as the 2022 European LeukemiaNet prognostication system (3).

The study by Sun et al. from Peking University Institute of Hematology assessed the T cell repertoire from bone marrow specimens of patients with AML and healthy controls. Bone marrow specimens from patients with AML were found to have greater frequencies of CD8(+) T cells. Patients with *DNMT3A* mutation had lower levels of CD8(+) T cells but higher levels of central memory T cells. Inferior outcomes were noted for patients with low memory T cell and CD8(+) T cell frequencies. This study provides insight into the correlation between mutational status and T cell subsets in AML. The T cell microenvironment at diagnosis may have a prognostic impact in AML, and the study merits further investigations into pro-tumor and anti-tumor immune surveillance mechanisms which may be at play in patients with myeloid neoplasms. Furthermore, knowledge of the immunologic landscape of AML may help pave way for identification of therapeutic targets.

The review by Jiang et al. from Zhengzhou University discusses the CD47/SIRPα interaction as a macrophage inhibitory checkpoint in AML. This axis was originally described in AML by Majeti et al. and Jaiswal et al. in 2009, when it was shown that CD47 was an adverse prognostic marker and therapeutic target in AML (4, 5). Jiang et al. provide us with an up-to-date summary of the Phase 3 ENHANCE trial of the anti-CD47 antibody magrolimab for high-risk MDS and highlight the challenges of translating these therapeutics, including SIRPα fusion proteins. Importantly, the CD47/SIRPα axis plays a role in *TP53*-mutant AML, which is arguably the most pressing need in the field, and the investigational pipeline would benefit from invigoration with promising candidate therapeutics (6, 7).

Finally, the mini-review by Kannan et al. discusses the basic biology of the immune microenvironment of the bone marrow of patients with MDS. The authors emphasize the range of therapeutic strategies and recent discoveries in MDS with a focus on emerging immunotherapeutics. They highlight knowledge gaps and offer solutions to moving the field forward.

The 5 articles in our Research Topic span the breadth of translational science related to the immunobiology of MDS and AML, from basic immunobiology to the clinical application of candidate therapeutics. A comprehensive understanding of the immunobiology of MDS and AML can help shed light into rational therapeutic strategies in the coming years. Bench-to-bedside success in this space often requires sound laboratory science that can translate into meaningful clinical investigative efforts. As the topic editors of this Research Topic, we propose a call to action for further multi-institutional collaborative efforts such that our field can make positive progress to help enhance the lives of the thousands of patients worldwide suffering from MDS and AML. The field of myeloid malignancies is beginning to embark upon new avenues in the setting of immune-based therapeutics, and we may see these efforts come to fruition within the next few years.
